# Supplementary material for: Childhood obesity is linked to putative neuroinflammation in brain white matter, hypothalamus, and striatum
Source: Cereb Cortex Commun. 2023 May 2;4(2):tgad007. doi: 10.1093/texcom/tgad007 (PMC10191798; doi:10.1093/texcom/tgad007)
Supplement: CCC_2022_00043_accept_supplement_PDF_tgad007 [file ccc_2022_00043_accept_supplement_pdf_tgad007.pdf]

Supplementary material for: Childhood obesity is linked to putative neuroinflammation in brain white matter, hypothalamus, and striatum

Zhaolong Li, BA; Amjad Samara, MD; Mary Katherine Ray, PhD; Jerrel Rutlin, BS; Cyrus A. Raji, MD, PhD; Joshua S. Shimony, MD, PhD; Peng Sun, PhD; Sheng-Kwei Song, PhD; Tamara Hershey, PhD*; Sarah A. Eisenstein, PhD

***Corresponding author:**
Tamara Hershey, PhD
James S. McDonnell Professor of Cognitive Neuroscience
Department of Psychiatry and Mallinckrodt Institute of Radiology
Washington University in St. Louis School of Medicine
4525 Scott Ave., MSC 8134-0070-02
St. Louis, MO 63110
Phone: (314)-362-5593
Email: tammy@wustl.edu

Table of content (in order of appearance in main text):

Page	Designation	Description
1	**Supplementary Fig. S1**	Participant selection flowchart
2	**Supplementary Methods**	Power analysis for detecting obesity-related microstructural alterations; DBSI model specification
3	**Supplementary Fig. S2**	Assessment of automated hypothalamus segmentation
4	**Supplementary Table S1**	DBSI-FF and RF outliers in the striatum and hypothalamus
5-6	**Supplementary Fig. S3**	Distributions of obesity-related measures
7	**Supplementary Table S2**	Associations between striatal and hypothalamic DBSI metrics and obesity-related measures at baseline
8-9	**Supplementary Table S3**	Sample exploratory associations between demographics and baseline obesity-related measures
10	**Supplementary Fig. S4**	Association between baseline DBSI-RF in the hypothalamus and gain in WC over two years
11	**Supplementary Table S4**	Associations between baseline striatal and hypothalamic DBSI metrics and one-year change (Δ) in obesity-related measures
12	**Supplementary Table S5**	Associations between baseline striatal and hypothalamic DBSI metrics and two-year change (Δ) in obesity-related measures
13	**Supplementary Table S6**	Associations between striatal RSI-RNI and obesity-related measures at baseline
14	**Supplementary Fig. S5**	Association between baseline RSI-RNI in the nucleus accumbens and caudate nucleus and gain in WC over one year
15	**Supplementary Table S7**	Associations between baseline striatal RSI-RNI and one-year change (Δ) in obesity-related measures
16	**Supplementary Table S8**	Associations between baseline striatal RSI-RNI and two-year change (Δ) in obesity-related measures
17	**Supplementary Fig. S6**	Bivariate correlations between DBSI-RF and RSI-RNI in the striatum

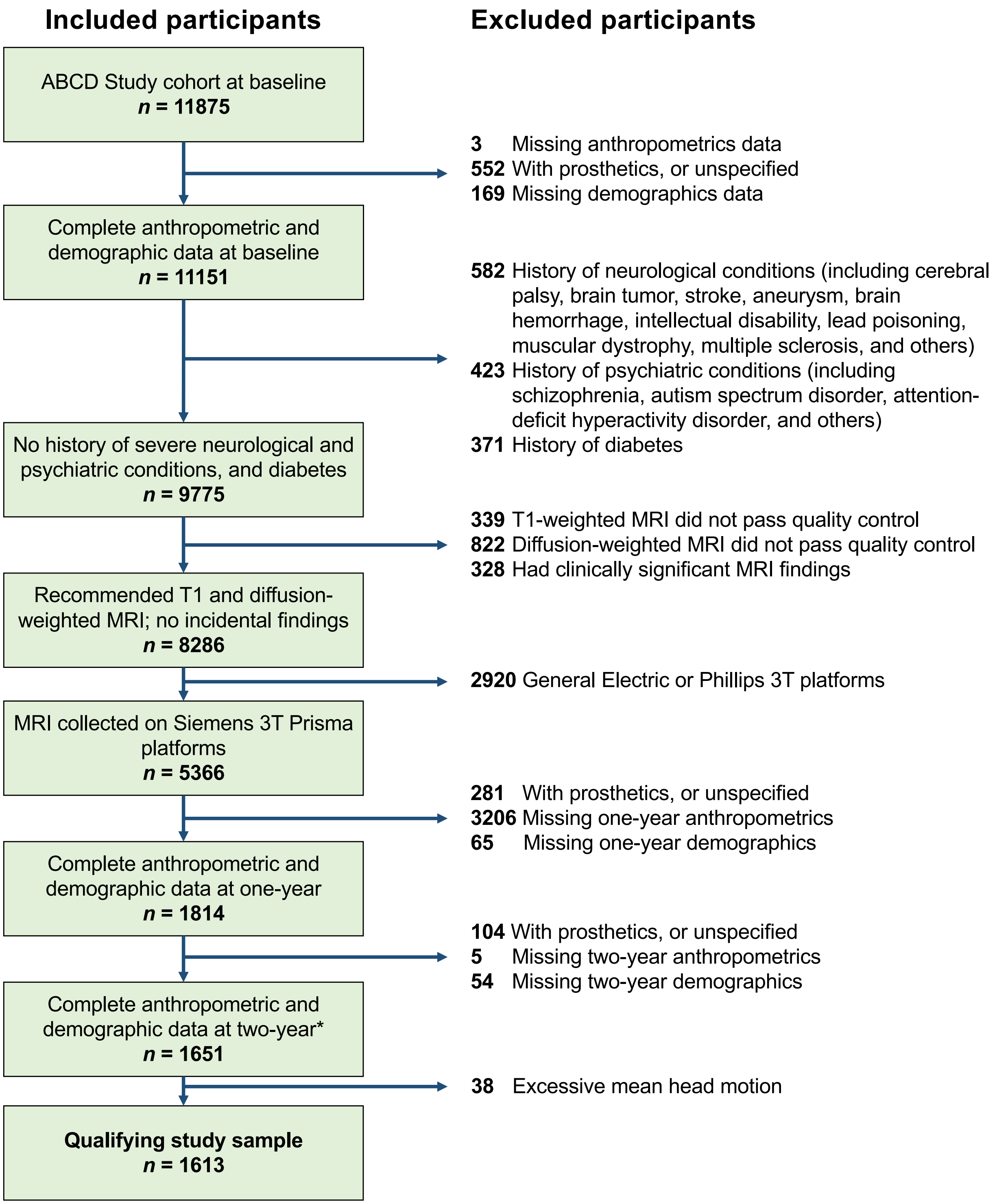

Supplementary Fig. 1. Participant selection flowchart. *Our study used data from the ABCD Study® 2.0.1 release, which included assessments for 11,875 participants at baseline and 4,951 participants at one-year follow-up. The inclusion/exclusion criteria here applied to the ABCD Study® 2.0.1 release. However, as the ABCD Study® 4.0 release became available near the end of our initially planned study, we included its two-year data to extend exploratory longitudinal analyses. ABCD, Adolescent Brain Cognitive Development; MRI, magnetic resonance imaging

Supplementary Methods

Power analysis. Data from 1,613 participants were qualified under our inclusion/exclusion criteria, of which 63 were with underweight, 1,140 were with normal-weight (NW), 194 were with overweight (OW), and 216 were with obesity (OB). To adequately sample for the spectrum of values of obesity-related measures, we processed data for all OW and OB participants, barring those with missing/incomplete/erroneous files. Processing raw DICOM images from the ABCD Study® 2.0.1 release involved time-consuming and computationally heavy steps that included preprocessing and segmenting T1-weighted images, distortion correction and diffusion tensor imaging (DTI) model-fitting for diffusion-weighted images, estimation of diffusion basis spectrum imaging (DBSI) maps, tract-based spatial statistics (TBSS), and quality control, which precluded the processing of all participants in a reasonable timeframe. To streamline analyses, we randomly selected 216 of the qualifying 1,140 NW participants stratified by sex, such that its groups size would be the same to that of the OB group. We reviewed the effect sizes for obesity-related alterations in brain microstructure, including DBSI measurements in white matter tracts and nucleus accumbens in adults (Cohen's d's ≥ 0.7) (Samara et al., 2020, 2021), restriction spectrum imaging (RSI) assessments in striatal regions in children (Cohen's d's ~ 0.5) (Rapuano et al., 2020), and quantitative T2-weighted magnetic resonance imaging (MRI) assessments in the hypothalamus in children and adults (Cohen's d's ≥ 0.65) (Sewaybricker et al., 2019; Sewaybricker, Kee, et al., 2021; Sewaybricker, Melhorn, et al., 2021). A power analysis based on these reported effect sizes indicated that a total sample size of 140 would provide 0.8 statistical power at $\alpha = 0.05$ to detect obesity-related microstructural alterations as assessed by DBSI. Our final analytical sample of $n = 601$ should thus have sufficient power. Further, such sample size is comparable to those in recent literature using the ABCD Study® data (Jiang et al., 2023; Sewaybricker, Kee, et al., 2021).

Diffusion basis spectrum imaging (DBSI) model specification. DBSI maps were estimated using in-house scripts as developed in Wang et al., (2011). Compared to traditional single-tensor DTI modeling, DBSI considers the total diffusion signal within each voxel to be a linear combination of multiple anisotropic tensors and a spectrum of isotropic diffusion, thereby resolving their confounding as is common in the imaging of neuroinflammation. The DBSI equation is specified below:

$$S_k = \sum_{i=1}^{N_{Aniso}} f_i e^{-|\overrightarrow{b_k}|.\lambda_{\perp_i}} e^{-|\overrightarrow{b_k}|.(\lambda_{\parallel_i}-\lambda_{\perp_i}).\cos^2\Phi_{ik}} + \int_a^b f(D) e^{-|\overrightarrow{b_k}|D} dD \; (k = 1,2,3,\ldots,k)$$

where S_k is the diffusion signal and b_k is the b-value of the kth diffusion gradient, N_{Aniso} is the number of anisotropic tensors to be determined, Φ_{ik} is the angle between the kth diffusion gradient and the principal direction of the ith anisotropic tensor, $\lambda_{\parallel_i}$ and $\lambda_{\perp_i}$ are the axial and radial water diffusivities of the ith anisotropic tensor, f_i is the signal intensity fraction for the ith anisotropic tensor, and a and b are low and high diffusivity limits of the isotropic diffusion spectrum $f(D)$.

DBSI further partitions the isotropic diffusion spectrum based on D cutoffs (i.e., diffusion scales) that empirically corresponded to water diffusion properties in different tissue microstructures, including isotropic nonrestricted fraction ($D > 0.3$ μm^2/ms; reflects vasogenic edema/tissue disintegration/extracellular water), and isotropic restricted fraction (DBSI-RF; $0 < D \leq 0.3$ μm^2/ms; reflects intracellular water/inflammation-related cellularity) (Wang et al., 2011, 2015). In the current study, DBSI-RF and one anisotropic metric, fiber fraction (DBSI-FF; reflects apparent axonal/dendritic density) served as assessments for brain microstructure. DBSI-FF and RF are consistently lower and higher, respectively, in adults with obesity (Samara et al., 2020, 2021).

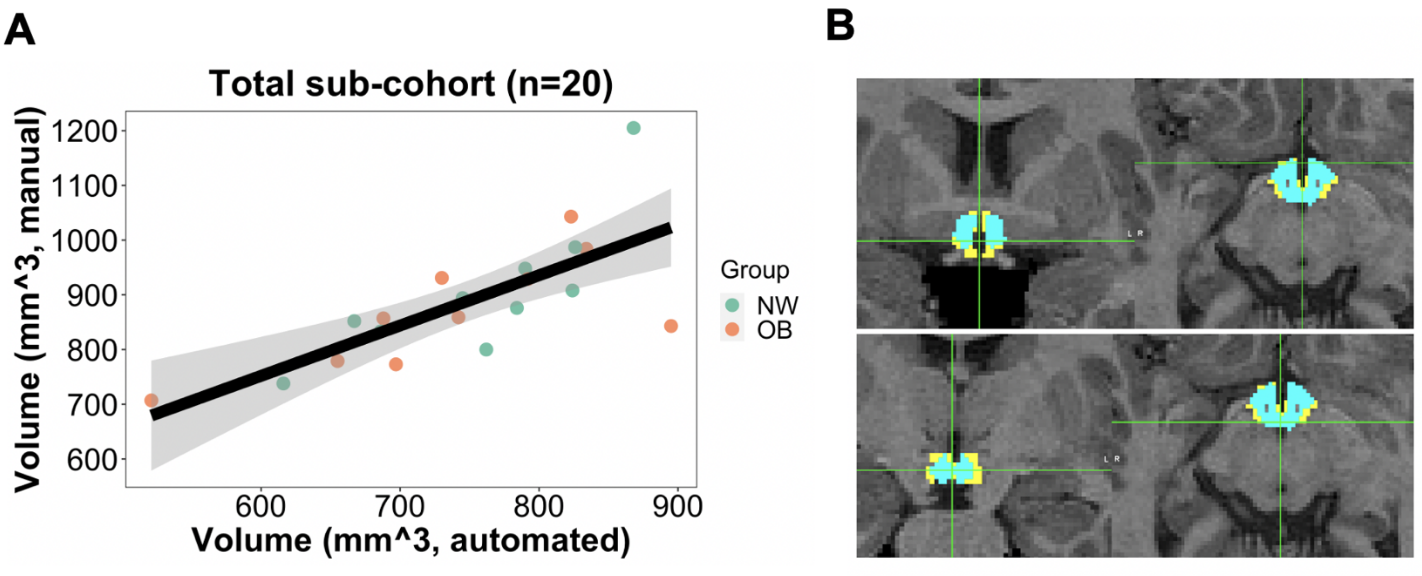

Supplementary Fig. 2. Assessment of automated hypothalamus segmentation. **(A)** Volumetric correlation of automated and manual hypothalamus segmentations in 10 randomly selected normal-weight (NW) and 10 randomly selected participants with obesity (OB). **(B)** Sample manual (in yellow) and automated (in blue) segmentations for an OB participant (*first row*, anterior slice in coronal (left) and axial (right) views; *second row*, posterior slice in coronal (left) and axial (right) views).

Region	Outlier counts		
	DBSI-FF	DBSI-RF	RSI-RNI
Hypothalamus	6 [1.00%]<br>1 NW, 2 OW, 3 OB	7 [1.16%]<br>2 NW, 3 OW, 2 OB	N/A
Nucleus accumbens	3 [0.50%]<br>1 OW, 2 OB	4 [0.67%]<br>2 NW, 1 OW, 1 OB	2 [0.33%]<br>1 NW, 1 OW
Caudate nucleus	3 [0.50%]<br>1 OW, 2 OB	9 [1.50%]<br>4 NW, 2 OW, 3 OB	7 [1.16%]<br>2 NW, 3 OW, 2 OB
Putamen	4 [0.67%]<br>2 OW, 2 OB	6 [1.00%]<br>1 NW, 1 OW, 4 OB	1 [0.17%]<br>1 OB

Supplementary Table 1. DBSI-FF and RF outliers in the striatum and hypothalamus. Outliers were defined as ± 3 standard deviations from the mean and were removed in analyses. Tallies are shown as count [frequency], followed by counts by weight groups. RSI-RNI data was not available for the hypothalamus. DBSI, diffusion basis spectrum imaging; FF, fiber fraction; RF, restricted fraction; RSI, restriction spectrum imaging; RNI, restricted normalized isotropic; NW, children with normal-weight; OW, children with overweight; OB, children with obesity.

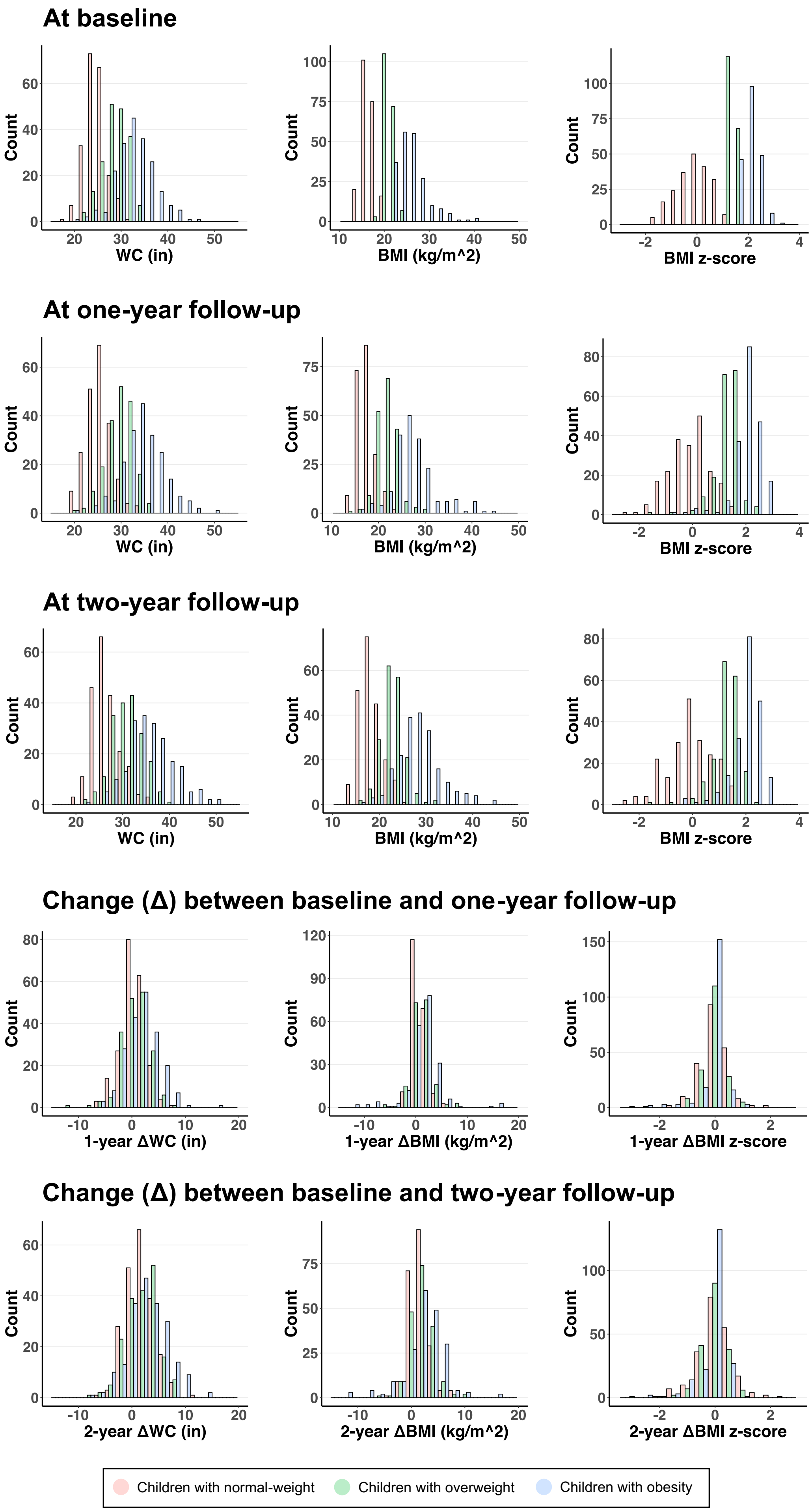
At baseline
At one-year follow-up
At two-year follow-up
Change (Δ) between baseline and one-year follow-up
Change (Δ) between baseline and two-year follow-up
Count
WC (in)
BMI (kg/m^2)
BMI z-score
1-year ΔWC (in)
1-year ΔBMI (kg/m^2)
1-year ΔBMI z-score
2-year ΔWC (in)
2-year ΔBMI (kg/m^2)
2-year ΔBMI z-score
Children with normal-weight
Children with overweight
Children with obesity

Supplementary Fig. 3. Distributions of obesity-related measures at baseline, one, and two-year follow-ups, and changes (Δ) between timepoints. $N = 601$ for 212 children with normal-weight, 187 with overweight, and 202 with obesity, except that extreme BMI values (< 10 kg/m^2 or > 50 kg/m^2) and corresponding BMI *z*-scores were removed for 1 participant with normal-weight and 1 participant with obesity at one-year and 1 participant with overweight at two-year. WC, waist circumference (in inches); BMI, body mass index (in kg/m^2).

Region	Imaging metric (predictor)	Baseline obesity-related measure (response)	*p*-value	Std. *β* (95% CI)	Partial R^2	N
**Hypothalamus**	DBSI-FF	WC	0.038	-0.08 [-0.15 to -0.01]	0.007	595
		BMI	0.024	-0.09 [-0.16 to -0.01]	0.009	595
		BMI *z*-score	**0.002**	-0.12 [-0.20 to -0.05]	0.016	595
	DBSI-RF	WC	0.011	0.10 [0.03 to 0.18]	0.012	594
		BMI	**0.005**	0.11 [0.04 to 0.19]	0.014	594
		BMI *z*-score	**0.004**	0.12 [0.04 to 0.20]	0.015	594
**Nucleus accumbens**	DBSI-FF	WC	0.009	-0.10 [-0.17 to -0.03]	0.011	598
		BMI	0.09	-0.07 [-0.14 to 0.01]	0.005	598
		BMI *z*-score	0.38	-0.03 [-0.11 to 0.04]	0.001	598
	DBSI-RF	WC	**< 0.001**	0.34 [0.25 to 0.42]	0.113	597
		BMI	**< 0.001**	0.30 [0.22 to 0.39]	0.096	597
		BMI *z*-score	**< 0.001**	0.26 [0.17 to 0.35]	0.070	597
**Caudate nucleus**	DBSI-FF	WC	0.11	-0.06 [-0.14 to 0.01]	0.004	598
		BMI	0.07	-0.07 [-0.15 to 0.01]	0.006	598
		BMI *z*-score	0.06	-0.07 [-0.15 to 0.00]	0.006	598
	DBSI-RF	WC	**< 0.001**	0.23 [0.13 to 0.33]	0.054	592
		BMI	**< 0.001**	0.18 [0.08 to 0.27]	0.034	592
		BMI *z*-score	**0.002**	0.15 [0.06 to 0.24]	0.023	592
**Putamen**	DBSI-FF	WC	0.14	-0.06 [-0.13 to 0.02]	0.004	597
		BMI	0.048	-0.08 [-0.16 to 0.00]	0.007	597
		BMI *z*-score	0.048	-0.08 [-0.16 to 0.00]	0.007	597
	DBSI-RF	WC	**< 0.001**	0.15 [0.06 to 0.23]	0.023	595
		BMI	**0.001**	0.14 [0.06 to 0.22]	0.021	595
		BMI *z*-score	0.015	0.11 [0.02 to 0.19]	0.012	595

Supplementary Table 2. Associations between striatal and hypothalamic DBSI metrics and obesity-related measures at baseline. Standardized (Std.) *β* regression coefficients were reported with 95% confidence intervals (CIs). Analyses were adjusted for age, sex, race/ethnicity, parental education, household income, parental marital status, pubertal development stage, mean head motion, intracranial volume, and family nested by site. Effects that survived multiple comparison corrected $p \leq 0.00625$ were considered statistically significant and are bolded. DBSI, diffusion basis spectrum imaging; FF, fiber fraction; RF, restricted fraction; WC, waist circumference; BMI, body mass index.

(A) Associations between hypothalamus DBSI-RF and baseline BMI (*n* = 594)

**Fixed effects**	**Unstandardized estimate**	**Standard error**	***p*-value**	**Partial R^2**
(Intercept)	6.5e+00	3.3e+00	0.051	
DBSI-RF	6.1e+01	2.2e+01	**0.005**	0.014
Age	5.1e-02	2.3e-02	**0.025**	0.009
Male sex	6.1e-01	4.6e-01	0.190	0.003
Black race/ethnicity (relative to White)	5.8e-01	6.1e-01	0.347	0.002
Hispanic race/ethnicity (relative to White)	9.7e-01	4.9e-01	**0.047**	0.008
Asian race/ethnicity (relative to White)	4.5e+00	1.6e+00	**0.006**	0.013
Other race/ethnicity (relative to White)	8.2e-01	6.3e-01	0.191	0.003
Parental education < HS (relative to Bachelor’s)	1.6e+00	1.8e+00	0.379	0.001
Parental education = HS/GED (relative to Bachelor’s)	1.7e+00	8.0e-01	**0.032**	0.008
Parental education = Some college (relative to Bachelor’s)	9.6e-01	5.2e-01	0.064	0.006
Parental education = Postgraduate (relative to Bachelor’s)	-5.4e-01	4.1e-01	0.187	0.003
Income < 50k (relative to >100k)	5.3e-01	4.1e-01	0.204	0.001
Income = 50-100k (relative to >100k)	3.4e-01	5.8e-01	0.562	0.003
Parents not married	3.7e-01	4.3e-01	0.391	0.001
PDS = 2 (relative to PDS = 1)	7.8e-01	4.1e-01	0.057	0.006
PDS = 3+ (relative to PDS = 1)	2.8e+00	5.4e-01	**4e-07**	0.042
Mean head motion	9.4e-02	5.9e-01	0.875	< 0.001
ICV	2.6e-06	1.3e-06	0.055	0.006
Total model				0.179

(B) Associations between caudate nucleus DBSI-RF and baseline BMI (*n* = 592)

**Fixed effects**	**Unstandardized estimate**	**Standard error**	***p*-value**	**Partial R^2**
(Intercept)	4.8e+00	3.4e+00	0.159	
DBSI-RF	2.0e+02	5.2e+01	**< 0.001**	0.034
Age	5.5e-02	2.3e-02	**0.017**	0.010
Male sex	8.1e-01	4.7e-01	0.083	0.005
Black race/ethnicity (relative to White)	1.0e+00	6.2e-01	0.103	0.005
Hispanic race/ethnicity (relative to White)	8.5e-01	4.9e-01	0.086	0.006
Asian race/ethnicity (relative to White)	3.9e+00	1.6e+00	**0.016**	0.010
Other race/ethnicity (relative to White)	8.5e-01	6.3e-01	0.179	0.003
Parental education < HS (relative to Bachelor’s)	1.9e+00	1.8e+00	0.304	0.002
Parental education = HS/GED (relative to Bachelor’s)	1.8e+00	8.1e-01	**0.030**	0.008
Parental education = Some college (relative to Bachelor’s)	1.3e+00	5.1e-01	**0.010**	0.011
Parental education = Postgraduate (relative to Bachelor’s)	-4.8e-01	4.1e-01	0.234	0.002
Income < 50k (relative to >100k)	5.8e-01	4.1e-01	0.159	< 0.001
Income = 50-100k (relative to >100k)	2.1e-01	5.8e-01	0.714	0.003
Parents not married	2.7e-01	4.3e-01	0.529	0.001
PDS = 2 (relative to PDS = 1)	7.7e-01	4.1e-01	0.065	0.005
PDS = 3+ (relative to PDS = 1)	2.8e+00	5.4e-01	**3.78e-07**	0.042

Mean head motion	-1.6e-01	6.2e-01	0.798	< 0.001
ICV	2.0e-06	1.3e-06	0.139	0.004
Total model				0.200

Supplementary Table 3. Sample exploratory associations between demographics and baseline obesity-related measures. Linear mixed-effects model outputs for associations between baseline body mass index (BMI) and DBSI-RF in the **(A)** hypothalamus and **(B)** caudate nucleus. Similar outputs were seen with BMI *z*-scores and waist circumference, and in other brain regions. Besides DBSI metrics, variables that showed associations with greater obesity-related measures included older age, lower parental education, and more advanced pubertal development stage (PDS). Although there were significant effects of Asian race/ethnicity, its relatively small group size (n = 1, 1, and 4 in normal-weight, overweight, and obesity groups; see **Table 1**) precludes reliable statistical inference. These exploratory results suggest a relationship between demographics and obesity that was controlled for but not tested in the current study. Effects that were nominally significant at $p \leq 0.05$ were bolded. DBSI, diffusion basis spectrum imaging; RF, restricted fraction; HS, high school; GED, General Education Development; ICV, intracranial volume.

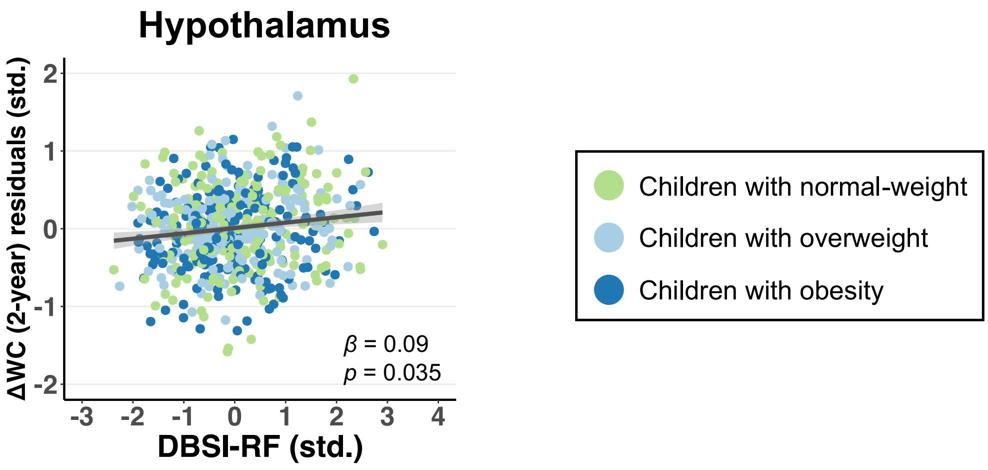

Supplementary Fig. 4. Association between baseline DBSI-RF in the hypothalamus and gain in waist circumference (WC) over two years. WC change (Δ) residuals (adjusted for baseline WC, age at two-year, sex, race/ethnicity, parental education, household income, parental marital status, pubertal development stage at two-year, mean head motion, intracranial volume, and family nested by site) and DBSI-RF were standardized (std.). Standardized β regression coefficients were reported with 95% confidence intervals (shaded). DBSI, diffusion basis spectrum imaging; RF, restricted fraction.

Region	Imaging metric (predictor)	1-year Δobesity-related measure (response)	*p*-value	Std. *β* (95% CI)	Partial R^2	N
**Hypothalamus**	DBSI-FF	WC	0.63	-0.02 [-0.10 to 0.06]	< 0.001	595
		BMI	0.34	0.04 [-0.04 to 0.13]	0.002	593
		BMI *z*-score	0.89	0.01 [-0.08 to 0.09]	< 0.001	593
	DBSI-RF	WC	0.26	0.05 [-0.04 to 0.13]	0.002	594
		BMI	0.97	-0.01 [-0.09 to 0.07]	< 0.001	592
		BMI *z*-score	0.49	0.03 [-0.06 to 0.11]	0.001	592
**Nucleus accumbens**	DBSI-FF	WC	0.89	-0.01 [-0.09 to 0.08]	< 0.001	598
		BMI	0.74	0.01 [-0.06 to 0.09]	< 0.001	596
		BMI *z*-score	0.95	0.00 [-0.08 to 0.08]	< 0.001	596
	DBSI-RF	WC	0.65	0.02 [-0.07 to 0.12]	< 0.001	597
		BMI	0.88	0.01 [-0.09 to 0.08]	< 0.001	595
		BMI *z*-score	0.85	0.01 [-0.08 to 0.09]	< 0.001	595
**Caudate nucleus**	DBSI-FF	WC	0.30	0.04 [-0.04 to 0.013]	0.002	598
		BMI	0.75	0.01 [-0.07 to 0.10]	< 0.001	596
		BMI *z*-score	0.59	0.02 [-0.06 to 0.10]	< 0.001	596
	DBSI-RF	WC	0.47	0.04 [-0.07 to 0.14]	0.001	592
		BMI	0.75	-0.02 [-0.11 to 0.06]	< 0.001	590
		BMI *z*-score	0.49	-0.03 [-0.11 to 0.05]	0.001	590
**Putamen**	DBSI-FF	WC	0.55	-0.03 [-0.11 to 0.06]	0.001	597
		BMI	0.21	0.05 [-0.03 to 0.14]	0.003	595
		BMI *z*-score	0.20	0.06 [-0.03 to 0.14]	0.003	595
	DBSI-RF	WC	0.53	0.03 [-0.06 to 0.12]	0.001	595
		BMI	0.29	-0.05 [-0.13 to 0.03]	0.002	593
		BMI *z*-score	0.76	-0.01 [-0.10 to 0.07]	< 0.001	593

Supplementary Table 4. Associations between baseline striatal and hypothalamic DBSI metrics and one-year change (Δ) in obesity-related measures. Standardized (Std.) *β* regression coefficients were reported with 95% confidence intervals (CIs). Analyses were adjusted for baseline obesity-related measures, age at one-year, sex, race/ethnicity, parental education, household income, parental marital status, pubertal development stage at one-year, mean head motion, intracranial volume, and family nested by site. DBSI, diffusion basis spectrum imaging; FF, fiber fraction; RF, restricted fraction; WC, waist circumference; BMI, body mass index.

Region	Imaging metric (predictor)	2-year Δobesity-related measure (response)	*p*-value	Std. *β* (95% CI)	Partial R^2	N
**Hypothalamus**	DBSI-FF	WC	0.17	-0.06 [-0.14 to 0.02]	0.003	595
		BMI	0.55	0.03 [-0.06 to 0.11]	0.001	594
		BMI *z*-score	0.72	-0.02 [-0.10 to 0.07]	< 0.001	594
	DBSI-RF	WC	0.035	0.09 [0.01 to 0.18]	0.008	594
		BMI	0.79	-0.01 [-0.10 to 0.07]	< 0.001	593
		BMI *z*-score	0.52	0.03 [-0.07 to 0.11]	0.001	593
**Nucleus accumbens**	DBSI-FF	WC	0.32	0.04 [-0.04 to 0.12]	0.002	598
		BMI	0.44	0.03 [-0.05 to 0.11]	0.001	597
		BMI *z*-score	0.64	0.02 [-0.06 to 0.10]	< 0.001	597
	DBSI-RF	WC	0.93	0.01 [-0.09 to 0.10]	< 0.001	597
		BMI	0.33	-0.05 [-014 to 0.04]	0.002	596
		BMI *z*-score	0.54	-0.03 [-0.12 to 0.05]	0.001	596
**Caudate nucleus**	DBSI-FF	WC	0.21	0.05 [-0.03 to 0.14]	0.003	598
		BMI	0.27	0.05 [-0.03 to 0.13]	0.002	597
		BMI *z*-score	0.30	0.04 [-0.04 to 0.13]	0.002	597
	DBSI-RF	WC	0.86	0.01 [-0.09 to 0.11]	< 0.001	592
		BMI	0.10	-0.08 [-0.17 to 0.01]	0.005	591
		BMI *z*-score	0.06	-0.08 [-0.16 to 0.00]	0.006	591
**Putamen**	DBSI-FF	WC	0.41	0.04 [-0.05 to 0.12]	0.001	597
		BMI	0.18	0.06 [-0.03 to 0.14]	0.003	596
		BMI *z*-score	0.09	0.07 [-0.01 to 0.16]	0.005	596
	DBSI-RF	WC	0.34	0.05 [-0.05 to 0.14]	0.002	595
		BMI	0.11	-0.07 [-0.16 to 0.01]	0.005	594
		BMI *z*-score	0.22	-0.05 [-0.14 to 0.03]	0.005	594

Supplementary Table 5. Associations between baseline striatal and hypothalamic DBSI metrics and two-year change (Δ) in obesity-related measures. Standardized (Std.) *β* regression coefficients were reported with 95% confidence intervals (CIs). Analyses were adjusted for baseline obesity-related measures, age at two-year, sex, race/ethnicity, parental education, household income, parental marital status, pubertal development stage at two-year, mean head motion, intracranial volume, and family nested by site. DBSI, diffusion basis spectrum imaging; FF, fiber fraction; RF, restricted fraction; WC, waist circumference; BMI, body mass index.

Region	Imaging metric (predictor)	Baseline obesity-related measure (response)	*p*-value	Std. *β* (95% CI)	Partial R^2	N
**Nucleus accumbens**	RSI-RNI	WC	< 0.001	0.38 [0.30 to 0.46]	0.138	599
		BMI	< 0.001	0.36 [0.27 to 0.44]	0.125	599
		BMI *z*-score	< 0.001	0.32 [0.24 to 0.40]	0.101	599
**Caudate nucleus**	RSI-RNI	WC	< 0.001	0.17 [0.09 to 0.25]	0.031	594
		BMI	< 0.001	0.15 [0.07 to 0.23]	0.025	594
		BMI *z*-score	0.001	0.13 [0.05 to 0.21]	0.019	594
**Putamen**	RSI-RNI	WC	< 0.001	0.18 [0.10 to 0.27]	0.034	600
		BMI	< 0.001	0.17 [0.09 to 0.26]	0.030	600
		BMI *z*-score	0.001	0.14 [0.06 to 0.22]	0.020	600

Supplementary Table 6. Associations between striatal RSI-RNI and obesity-related measures at baseline. Standardized (Std.) *β* regression coefficients were reported with 95% confidence intervals (CIs). Analyses were adjusted for age, sex, race/ethnicity, parental education, household income, parental marital status, pubertal development stage, mean head motion, intracranial volume, and family nested by site. RSI, restriction spectrum imaging; RNI, restricted normalized isotropic; WC, waist circumference; BMI, body mass index.

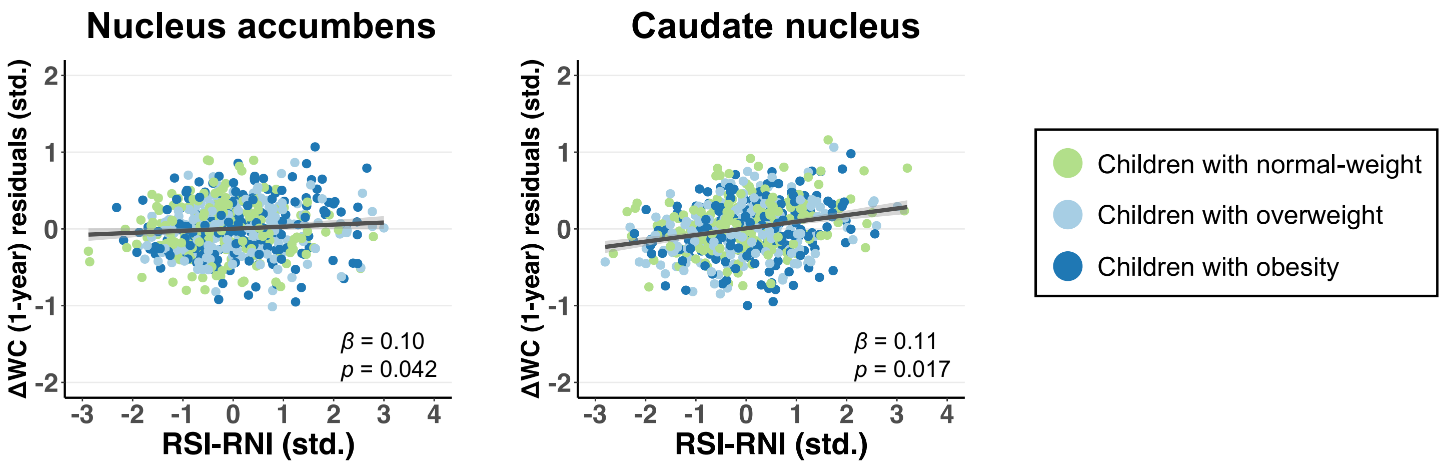

Supplementary Fig. 5. Association between baseline RSI-RNI in the nucleus accumbens and caudate nucleus and gain in waist circumference (WC) over one year. WC change (Δ) residuals (adjusted for baseline WC, age at one-year, sex, race/ethnicity, parental education, household income, parental marital status, pubertal development stage at one-year, mean head motion, intracranial volume, and family nested by site) and RSI-RNI were standardized (std.). Standardized β regression coefficients were reported with 95% confidence intervals (shaded). RSI, restriction spectrum imaging; RNI, restricted normalized isotropic.

Region	Imaging metric (predictor)	1-year Δobesity-related measure (response)	*p*-value	Std. *β* (95% CI)	Partial R^2	N
**Nucleus accumbens**	RSI-RNI	WC	0.042	0.10 [0.00 to 0.20]	0.008	599
		BMI	0.22	0.06 [-0.05 to 0.16]	0.003	597
		BMI *z*-score	0.33	0.05 [-0.06 to 0.14]	0.002	597
**Caudate nucleus**	RSI-RNI	WC	0.017	0.11 [0.02 to 0.19]	0.011	594
		BMI	0.15	0.06 [-0.03 to 0.15]	0.004	592
		BMI *z*-score	0.66	0.02 [-0.07 to 0.10]	< 0.001	592
**Putamen**	RSI-RNI	WC	0.14	0.07 [-0.02 to 0.17]	0.004	600
		BMI	0.49	0.03 [-0.06 to 0.12]	0.001	598
		BMI *z*-score	0.38	0.04 [-0.05 to 0.13]	0.001	598

Supplementary Table 7. Associations between baseline striatal RSI-RNI and one-year change (Δ) in obesity-related measures. Standardized (Std.) *β* regression coefficients were reported with 95% confidence intervals (CIs). Analyses were adjusted for baseline obesity-related measures, age at one-year, sex, race/ethnicity, parental education, household income, parental marital status, pubertal development stage at one-year, mean head motion, intracranial volume, and family nested by site. RSI, restriction spectrum imaging; RNI, restricted normalized isotropic; WC, waist circumference; BMI, body mass index.

Region	Imaging metric (predictor)	2-year Δobesity-related measure (response)	*p*-value	Std. *β* (95% CI)	Partial R^2	N
**Nucleus accumbens**	RSI-RNI	WC	0.10	0.08 [-0.02 to 0.18]	0.005	599
		BMI	0.30	0.05 [-0.05 to 0.15]	0.002	598
		BMI *z*-score	0.26	0.06 [-0.05 to 0.15]	0.002	598
**Caudate nucleus**	RSI-RNI	WC	0.24	0.05 [-0.03 to 0.14]	0.003	594
		BMI	0.36	0.04 [-0.05 to 0.13]	0.001	593
		BMI *z*-score	0.77	0.01 [-0.08 to 0.10]	< 0.001	593
**Putamen**	RSI-RNI	WC	0.47	0.04 [-0.06 to 0.13]	0.001	600
		BMI	0.81	0.01 [-0.08 to 0.10]	< 0.001	599
		BMI *z*-score	0.89	-0.01 [-0.10 to 0.08]	< 0.001	599

Supplementary Table 8. Associations between baseline striatal RSI-RNI and two-year change (Δ) in obesity-related measures. Standardized (Std.) *β* regression coefficients were reported with 95% confidence intervals (CIs). Analyses were adjusted for baseline obesity-related measures, age at two-year, sex, race/ethnicity, parental education, household income, parental marital status, pubertal development stage at two-year, mean head motion, intracranial volume, and family nested by site. RSI, restriction spectrum imaging; RNI, restricted normalized isotropic; WC, waist circumference; BMI, body mass index.

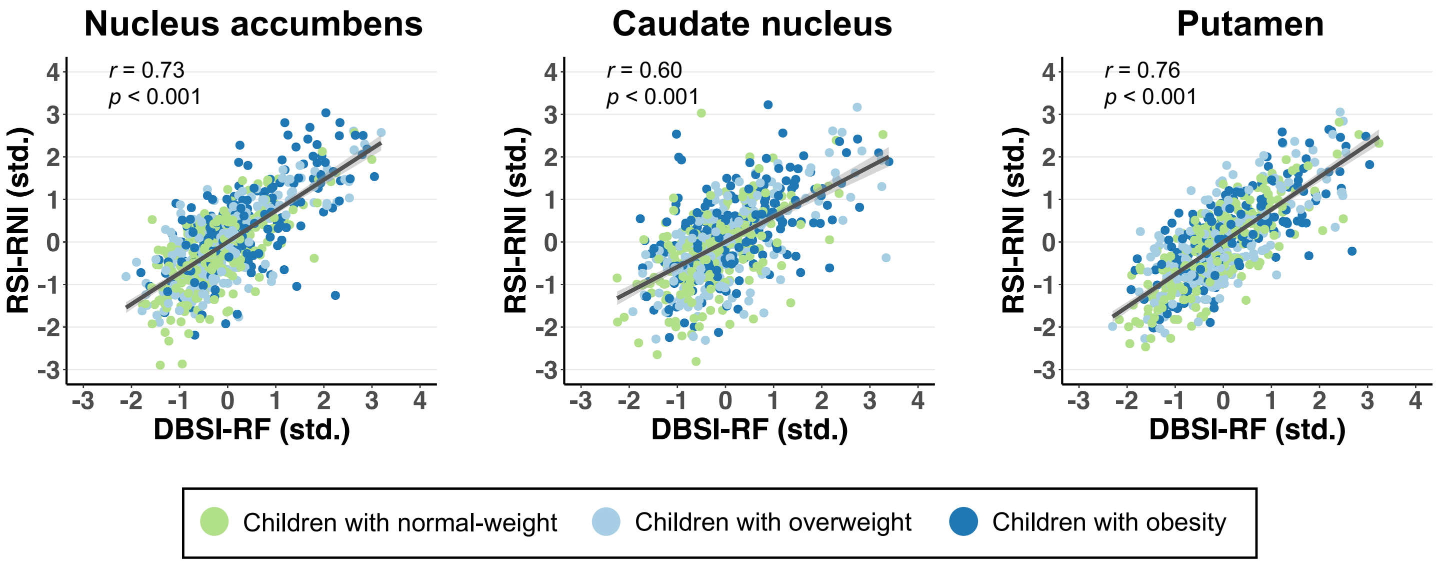

Supplementary Fig. 6. Bivariate correlations between DBSI-RF and RSI-RNI in the striatum. Shaded regions represent 95% confidence interval. Correlations were reported as Pearson's *r* coefficients.
